# AlGaN/GaN MISHEMTs with AlN gate dielectric grown by thermal ALD technique

**DOI:** 10.1186/s11671-015-0802-x

**Published:** 2015-03-04

**Authors:** Xiao-Yong Liu, Sheng-Xun Zhao, Lin-Qing Zhang, Hong-Fan Huang, Jin-Shan Shi, Chun-Min Zhang, Hong-Liang Lu, Peng-Fei Wang, David Wei Zhang

**Affiliations:** State Key Laboratory of ASIC and System, School of Microelectronics, Fudan University, 220 Han Dan Road, Shanghai, 200433 China

**Keywords:** AlGaN/GaN HEMT, 2DEG, ALD, MISHEMT

## Abstract

Recently, AlN plasma-enhanced atomic layer deposition (ALD) passivation technique had been proposed and investigated for suppressing the dynamic on-resistance degradation behavior of high-electron-mobility transistors (HEMTs). In this paper, a novel gate dielectric and passivation technique for GaN-on-Si AlGaN/GaN metal-insulator-semiconductor high-electron-mobility transistors (MISHEMTs) is presented. This technique features the AlN thin film grown by thermal ALD at 400°C without plasma enhancement. A 10.6-nm AlN thin film was grown upon the surface of the HEMT serving as the gate dielectric under the gate electrode and as the passivation layer in the access region at the same time. The MISHEMTs with thermal ALD AlN exhibit enhanced on/off ratio, reduced channel sheet resistance, reduction of gate leakage by three orders of magnitude at a bias of 4 V, reduced threshold voltage hysteresis of 60 mV, and suppressed current collapse degradation.

## Background

AlGaN/GaN-based high-electron-mobility transistors (HEMTs) are capable of delivering excellent performance such as high electron mobility, high saturation current, low on-resistance, and large breakdown voltage, all of which make AlGaN/GaN HEMTs suitable for RF and power applications [1-3]. However, the conventional AlGaN/GaN HEMTs using Schottky gates suffer from low turn-on voltage due to low Schottky barrier between gate metal and AlGaN. On the other hand, the potential of AlGaN/GaN HEMTs is greatly limited for high-speed and high-power applications by the current collapse effect. This effect is also described as dynamic behavior degradation, gate lag, drain lag, etc., which is caused by the charge trapping effect on the AlGaN surface especially in the access region between gate and drain [4-6]. In order to solve these two issues, the deposition of oxide-based dielectric such as SiO_2_ [7], Al_2_O_3_ [8], LaLuO_3_ [9], and SrO_2_ [10,11] and dielectric stack such as SiN/Al_2_O_3_ [12] as gate dielectric layer and passivation layer in the access region simultaneously is widely adopted. But it has been revealed that the introduction of oxide-based dielectric is likely to form Ga-O bond which is thought as one of interface sources leading to current collapse [13]. In this way, nitride-based dielectrics especially AlN are more favored due to the larger bandgap of AlN compared with that of SiN and smaller lattice mismatch.

Recently, AlN grown by plasma-enhanced atomic layer deposition (PEALD) as passivation layer has been proposed [14,15]. The thickness of AlN can be precisely controlled due to the nature of atomic layer deposition. Meanwhile, *in situ* surface pretreatment by ammonia (NH_3_) plasma can significantly suppress the dynamic on-resistance degradation especially under a large drain bias. AlN has also been used as an insertion layer between the Al_2_O_3_ and AlGaN to improve the interface [16,17]. However, the possible introduction of damage from plasma treatment may deteriorate the quality of film. Therefore, this work focuses on the thermal ALD AlN passivation method without plasma enhancement. Up to now, there are rare reports on AlN grown by thermal ALD for AlGaN/GaN HEMT passivation [18] and no report on thermal-grown AlN as gate dielectric of GaN HEMTs. The thermal ALD can deposit film with high crystallinity and results in good interface quality due to higher growth temperature. Compared with PEALD AlN using N_2_ as the source of ammonia in most reported AlGaN/GaN HEMT works, thermal ALD using NH_3_ instead of N_2_ can supply N-H more efficiently to remove carbon at the depositing surface [19].

In this letter, we show the thermal ALD AlN film serving as the gate dielectric and passivation technique without the assistance of plasma source. Many advantages such as reduction of gate leakage, improved low threshold voltage hysteresis, and suppressed current collapse degradation are obtained. A breakdown voltage of over 400 V with gate-drain spacing of 10 μm is obtained.

## Methods

**Figure 1:**
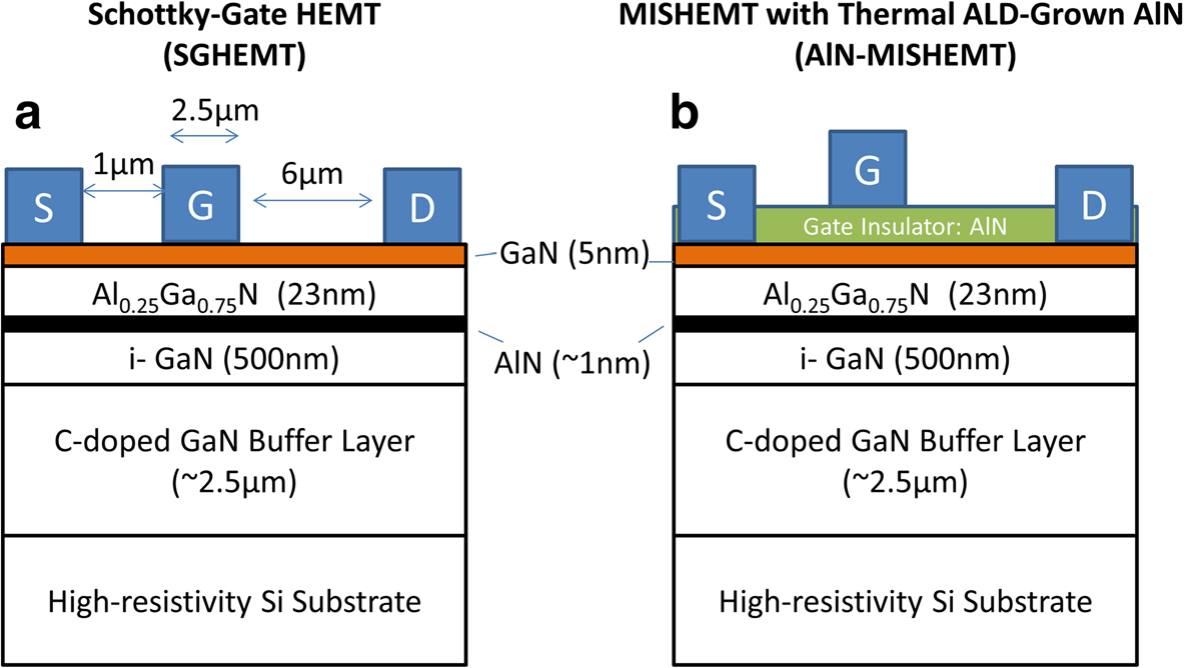
**Schematic cross section and dimensions of the HEMTs. (a)** Without AlN gate insulator and **(b)** with AlN gate insulator. These devices are with source-gate spacing *L*_GS_ of 1 μm, gate length *L*_G_ of 2.5 μm, drain-gate spacing *L*_GD_ of 6 μm, and gate width of 60 μm.

Figure 1a,b shows the schematic cross section of the Schottky gate HEMTs (SGHEMTs) and the AlN HEMTs (AlN-MISHEMTs) with a thermal ALD AlN gate insulator. The measured devices are with a source-gate spacing *L*_GS_ of 1 μm, gate length *L*_G_ of 2.5 μm, drain-gate spacing *L*_GD_ of 6 μm, and gate width of 60 μm.

**Figure 2:**
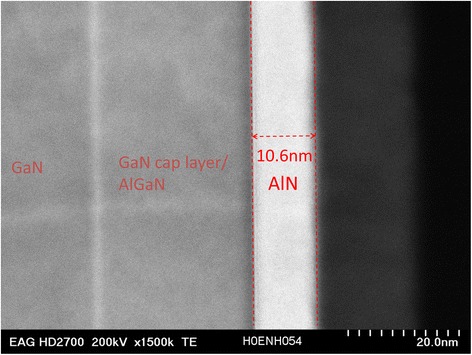
**HRTEM cross-sectional view of AlN-MISHEMT.**

In order to study the performance enhancement caused by incorporation of thermal ALD AlN through the comparison between the AlN-MISHEMTs and the SGHEMTs, those devices have an identical layout. The fabrication processes are almost the same except the AlN ALD process step before gate metal deposition. The samples are 2 cm × 2 cm square pieces sliced from the same 6-in GaN-on-Si wafer. The AlGaN/GaN heterostructure on the epi-wafer was grown by metalorganic chemical vapor deposition (MOCVD) on a high-resistivity silicon wafer and consists of 2.5-μm carbon-doped GaN buffer layer, 500-nm i-GaN layer, 1-nm AlN interlayer, 23-nm undoped Al_0.25_Ga_0.75_N top barrier layer, and 5-nm GaN cap layer. The sheet carrier density of 9.86 × 10^12^ cm^−2^, the two-dimensional electron gas (2DEG) mobility of 906 cm^2^/Vs, and channel sheet resistance of 700 Ω/sq were obtained by Hall measurement. In the first step, the mesas were defined by BCl_3_-based reactive-ion etching (RIE). Secondly, ohmic contacts were formed by e-beam evaporation and lift-off of Ti/Al/Ni/Au followed by a rapid thermal annealing at 850°C. The specific contact resistivity and contact resistance were 5.71 × 10^−5^ Ω · cm^2^ and 2 Ω · mm, respectively, obtained by transmission line measurement (TLM). Due to the variation of ohmic contact in the fabrication process, the specific contact resistivity and contact resistance of the AlN-MISHEMT sample are 1.81 × 10^−5^ Ω · cm^2^ and 1.27 Ω · mm, respectively. The AlN-HEMTs have an AlN gate dielectric layer deposited at a growth temperature of 400°C by using a BENEQ TFS 200 ALD reactor (Beneq Oy, Vantaa, Finland). Before AlN deposition, the surface native oxide was removed by dipping the samples in HCl:H_2_O (1:10) for 60 s. Trimethylaluminum (TMA) and NH_3_ have been utilized as aluminum and nitrogen sources, respectively. During all growth experiments, the NH_3_ gas flow rate was 100 sccm, exposure time was 0.9 s, TMA pulse time was 0.2 s, and purge time in between precursor pulses was 9 s. A total of 100-cycle AlN layers were deposited on the samples. The thickness was about 10.6 nm, measured in the TEM picture in Figure 2. The material composition property of the film is measured by XPS and will be published in the following paper. Atomic concentrations of Al, N, O, and C are 46.8%, 43.2%, 7.4%, and 2.6% respectively. The SGHEMTs omit this AlN thermal ALD step. Finally, Ni/Au gate electrodes were formed by e-beam evaporation and lift-off on both HEMTs.

## Results and discussion

**Figure 3:**
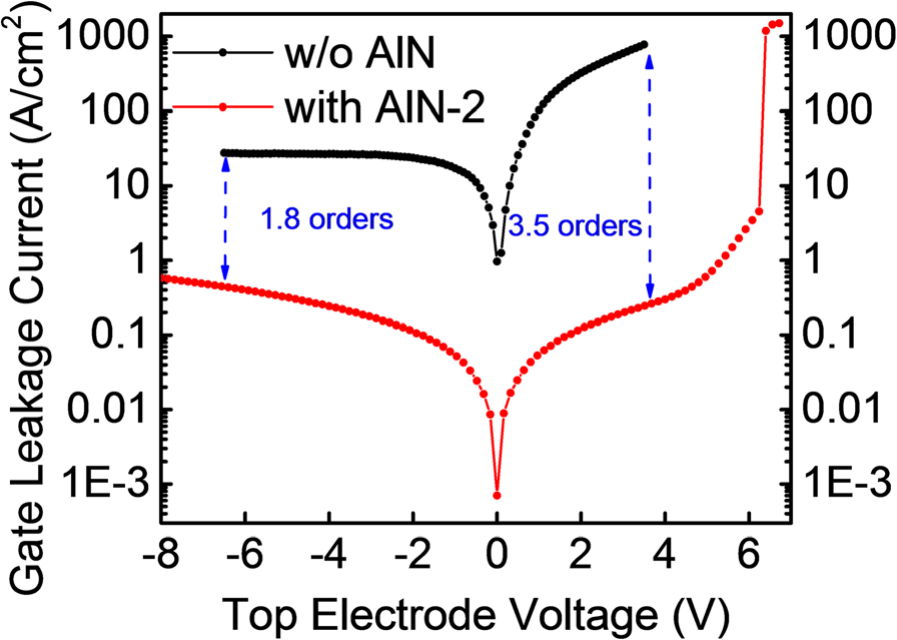
**Gate leakage current of Ni-Au/AlGaN-GaN Schottky diode and Ni-Au/AlN/AlGaN-GaN MIS diode.**

Circular Ni-Au/AlGaN-GaN Schottky diode and Ni-Au/AlN/AlGaN-GaN MIS diode are used to characterize the deposited film quality and the interface. Both diodes are with a diameter of 200 μm. The gate leakage current characteristics measured by Agilent 1500 semiconductor analyzer (Keysight Technologies, Inc., Englewood, CO, USA) are shown in Figure 3. With the insertion of thin film AlN gate insulator, the forward current can be reduced by three orders of magnitude at the bias of 4 V and the reverse current can be reduced by about one order of magnitude at the bias of −6 V. A forward gate bias voltage as high as 5.3 V can be reached if the limit of the gate leakage current density is 1 A/cm^2^.

**Figure 4:**
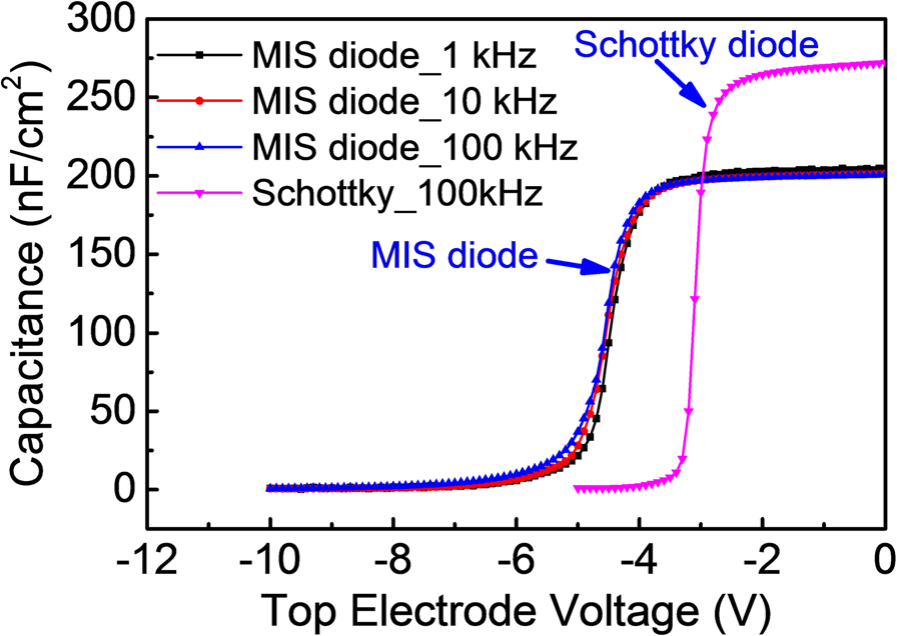
***C*** **-** ***V*** **characteristics of Ni-Au/AlGaN-GaN Schottky diode and Ni-Au/AlN/AlGaN-GaN MIS diode.**

Agilent 4294 precision impedance analyzer was used to measure the frequency-dependence capacitance-voltage characteristics of the MIS diodes and the Schottky diodes. In Figure 4, the *C*-*V* characteristics of the Ni-Au/AlGaN-GaN Schottky diode and the Ni-Au/AlN/AlGaN-GaN MIS diode are compared. A frequency dispersion of about 2.2% in the 2DEG accumulation region can be observed. The threshold voltage dispersion is negligible when the frequency is varied from 1 to 100 kHz. The capacitance of the Schottky and MIS diodes is 272 and 214 nF/cm^2^, respectively, in the accumulation region.

**Figure 5:**
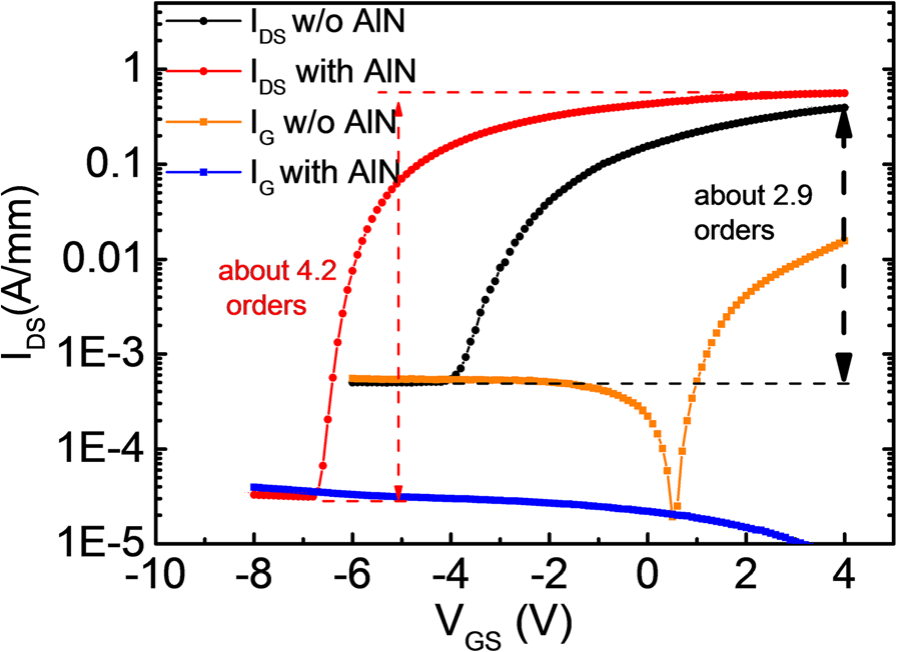
**Comparison of transfer characteristics of AlN-MISHEMT and SGHEMT with a drain voltage of 15 V.**

A comparison of the logarithm scale transfer curves of AlN-MISHEMT and SGHEMT at a drain voltage of 15 V is shown in Figure 5. The threshold voltage and the maximum transconductance *G*_m_ of AlN-MISHEMT and SGHEMT are −5.7 V and 87 mS/mm and −2.3 V and 70 mS/mm, respectively, determined by linear extrapolation of the transfer curve at the point of peak transconductance. Using AlN as the gate dielectric, AlN-MISHEMT demonstrates a drain current enhancement from 0.396 to 0.563 A/mm at a gate bias of 5 V and a transconductance enhancement from 70 to 87 mS/mm. The channel sheet resistance of AlN-MISHEMT measured from the TLM test structure became 573 Ω/sq, which is smaller than 700 Ω/sq of SGHEMT. That is because of the additional polarization charges caused by AlD AlN film [20]. The transconductance and drain current enhancement might be caused by the reduction of the gate-source and gate-drain series resistance in AlN-MISHEMT. In Figure 5, the off-state drain current of AlN-MISHEMT is also reduced by one order of magnitude at the gate bias of −8 V. Meanwhile, strong reduction in gate leakage current is simultaneously achieved. When these two devices are turned off, the drain leakage current is almost equal to the gate leakage current, which is a clear indication that the off-state drain current is mainly caused by the gate leakage in these devices. Because of the enhanced on-current and reduced leakage current, the on/off ratio is raised from 2.9 to 4.2 decades by using thermal ALD AlN as the gate insulator.

**Figure 6:**
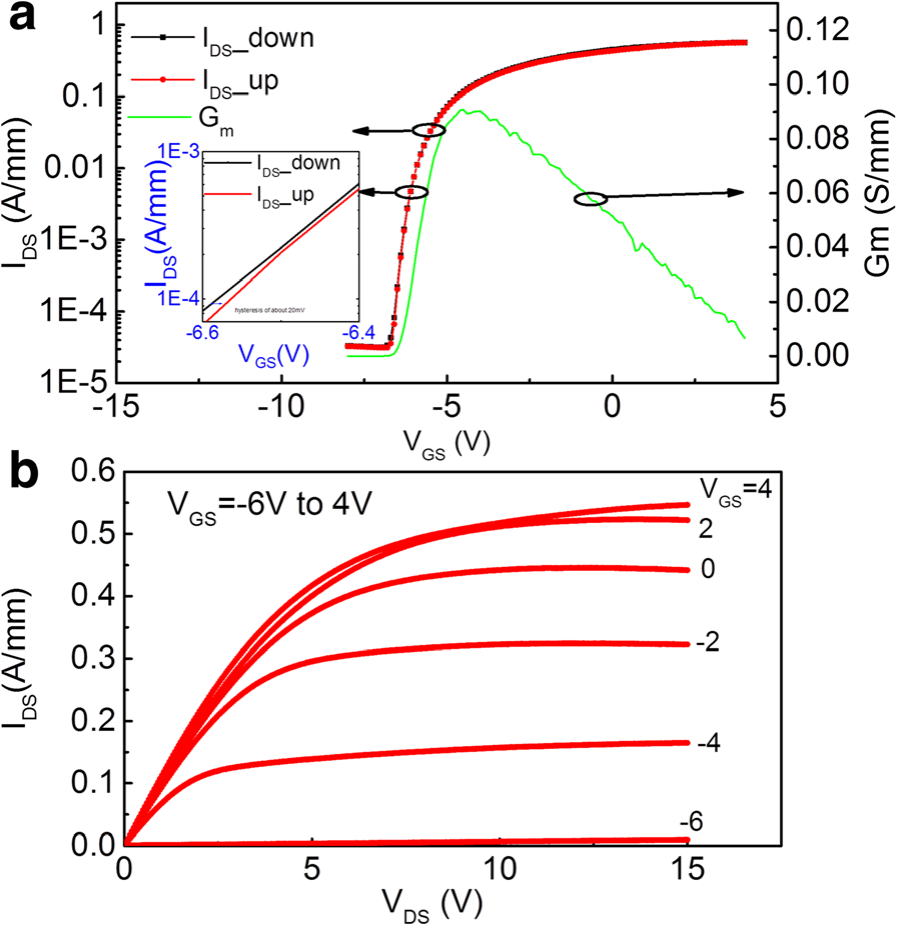
**Transfer and output characteristics of an AlN-MISHEMT. (a)** Forward-sweep and back-sweep transfer characteristics of AlN-MISHEMT; **(b)** output characteristics of AlN-MISHEMT.

The transfer and output characteristics of an AlN-MISHEMT are plotted in Figure 6a,b. In the transfer curves shown in Figure 6a, the gate bias is swept forward from −8 to 4 V and swept back from 4 to −8 V with a drain bias of 15 V. A very small threshold voltage hysteresis of 60 mV is observed between these two transfer curves, indicating the good interface between AlN and AlGaN. In Figure 6b, the gate voltage is varied from −6 to 4 V with 2 V/step. The drain current has a maximum drain current of 0.540 A/mm. The drain current may suffer from the electric field-related mobility degradation when the gate voltage is close to 4 V.

**Figure 7:**
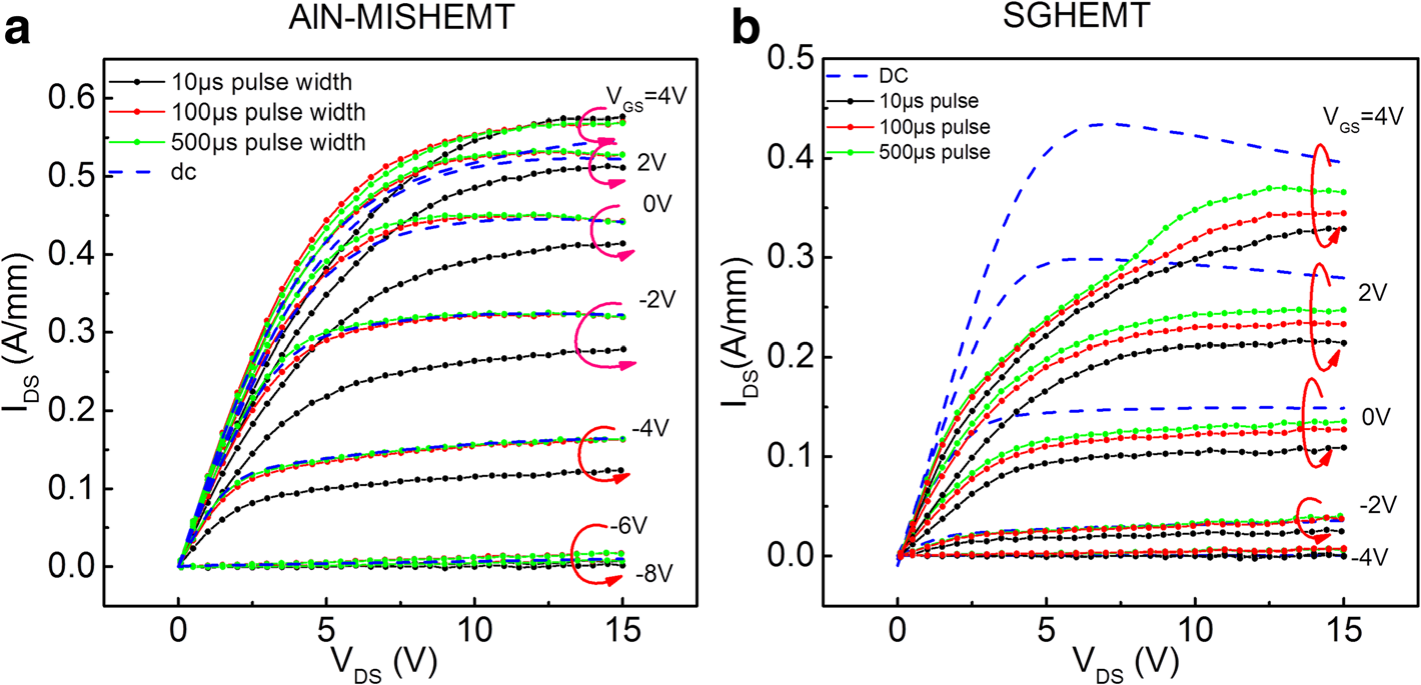
**DC and gate turn-on pulse-mode** ***I***_DS_ **-** ***V***_DS_ **characteristics (a) of AlN-MISHEMT and (b) of SGHEMT.**

To evaluate the degradation of the current collapse in SGHEMT and AlN-MISHEMT, Agilent 1525A signal pulse generator unit was used to perform turn-on pulse-mode measurement. The DC and gate turn-on pulse-mode *I*_DS_-*V*_DS_ characteristics of AlN-MISHEMT and SGHEMT are shown in Figure 7. The gate voltage was pulsed from threshold voltage to 2 V with three different pulse widths of 10, 100, and 500 μs. The drain was pulsed from 0 V to *V*_DS_ with a pulse width of 9 ms. The total pulse cycle is 10 ms. The current measurement was made at the peak of the gate pulse. As can be seen from Figure 7a, only a slight drain current degradation is observed in the 10-μs pulse measurement for AlN-MISHEMT. Almost no drain current degradation in 100- and 500-μs pulse measurements is observed for AlN-MISHEMT. For comparison, a large drain current collapse can be observed even with 500-μs pulse width in Figure 7b where SGHEMT measurement results are plotted. This comparison suggests that using thermal ALD, AlN can annihilate the surface states and effectively suppress the drain current collapse effect.

**Figure 8:**
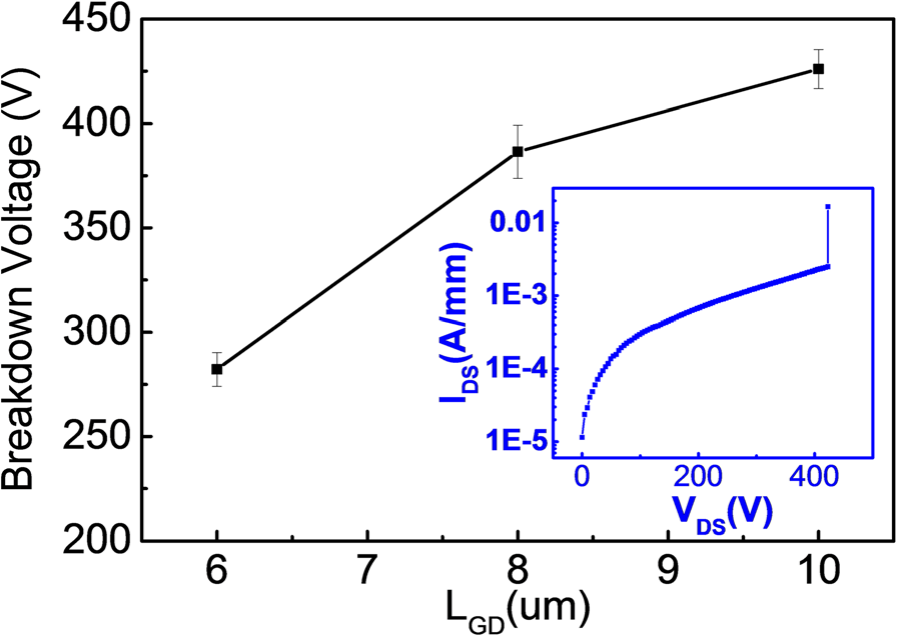
**Relationship between breakdown voltage and gate-drain spacing** ***L***_GD_ **.** Inset: three-terminal off-state breakdown characteristics of an AlN-MISHEMT with *L*_GS_ = 1 μm, *L*_G_ = 2.5 μm, and *L*_GD_ = 10 μm at *V*_GS_ = −7 V.

The three-terminal off-state breakdown characteristics of AlN-MISHEMTs with *L*_G_ = 2.5 μm, *L*_GS_ = 1 μm, and various *L*_GD_ were shown in Figure 8. The breakdown voltage is extracted at the point of *V*_DS_ when the drain current reaches 0.010 A/mm at *V*_GS_ = −7 V. The inset shows the measured breakdown characteristics of an AlN-MISHEMT with *L*_GD_ = 10 μm. The breakdown voltage is 280 V with *L*_GD_ = 6 μm and reaches 425 V with *L*_GD_ = 10 μm.

## Conclusions

The gate dielectric and passivation technique of GaN-HEMT using thermal ALD AlN without plasma enhancement is firstly reported in this letter. The enhanced DC and transient response performances of AlGaN/GaN MISHEMTs with thermal ALD AlN have been comprehensively investigated. With the insertion of thermal AlD AlN, the AlN-MISHEMT delivers a higher *I*_on_/*I*_off_ ratio of 10^4^ due to the reduced gate leakage current of 1 A/cm^2^ and very small threshold voltage hysteresis of 60 mV, indicating the good interface between AlN and AlGaN. The breakdown voltage of AlN-MISHEMTs is over 400 V with an *L*_GD_ of 10 μm. The thermal AlD-grown AlN also exhibits effective passivation and improved drain collapse effect. The thermal ALD AlN technique is therefore a very promising method to achieve high-performance GaN MISHEMT for RF and power applications.
